# Aerosol deposition in the lung as a biomarker in asthma and chronic obstruction

**DOI:** 10.1186/s12931-026-03706-2

**Published:** 2026-05-11

**Authors:** Hugo Öhrneman, Andrei Malinovschi, Gui-Hong Cai, Magnus Svartengren, Hanna Nicklasson, Jonas Jakobsson, Per Wollmer, Jakob Löndahl

**Affiliations:** 1 Division of Ergonomics and Aerosol Technology, Department of Design Sciences, Lund University, Box 118, Lund, 221 00 LUND Sweden; 2 Department of Medical Sciences, Clinical Physiology, Uppsala University, Uppsala, Sweden; 3 Department of Medical Sciences, Occupational and Environmental Medicine, Uppsala University, Uppsala, Sweden; 4 Department of Translational Medicine, Clinical Physiology and Nuclear Medicine, Lund University, Malmö, Sweden

**Keywords:** Chronic obstructive airway disease, Chronic airflow limitation, Asthma, Emphysema, Lung function, Aerosols

## Abstract

**Background:**

The objective of this study was to explore the potential of Airspace Dimension Assessment (AiDA) to rapidly determine the type and severity of airway obstruction, detect emphysema in individuals with chronic airflow limitation (CAL), and distinguish it from non-emphysematous obstruction, asthma, and absence of respiratory disease with normal spirometry.

**Methods:**

Among the 744 participants measured with AiDA within the Swedish CArdioPulmonary bioImage Study (SCAPIS) cohort, 40 had asthma, and 34 had a CAL (defined by a post-bronchodilator FEV_1_/FVC < 0.7), whereof 12 had CT-detected emphysema. AiDA measurements were compared across these groups and to 111 healthy controls (never-smokers with normal spirometry and no history of respiratory disease).

**Results:**

Subjects with CAL had significantly larger distal airspaces radii (median *r*_AiDA_=298 μm) than controls (*r*_AiDA_=278 μm, *p* < 0.001), but no significant difference was observed in asthmatics (*r*_AiDA_=273 μm, *p* = 0.79). Subjects with CT-detected emphysema in the CAL group displayed further differentiation from the control (*r*_AiDA_=349 μm, *p* < 0.001), while those without emphysema displayed no significant increase. Unlike *r*_AiDA_, neither low attenuation volume nor 15th percentile density could clearly distinguish between obstruction and radiologist-assessed emphysema. In addition, the zero-second particle recovery (*R*_0_), which is theorized to reflect conducting airway dysfunction, was decreased in both asthmatics (*R*_0_ = 0.41, *p* = 0.011), and in the CAL group (*R*_0_ = 0.45, *p* = 0.020) when compared to controls (*R*_0_ = 0.56).

**Conclusions:**

These findings display AiDA’s potential in identifying emphysema as well as obstructive airway disease. The absence of an increased distal airspace radius in asthmatics confirm that *r*_AiDA_ is a measure of the distal airspaces, unaffected by abnormalities in the conducting airways. However, the decreased *R*_0_ in both asthma and CAL suggests that *R*_0_ does reflect conducting airway abnormality.

**Supplementary Information:**

The online version contains supplementary material available at 10.1186/s12931-026-03706-2.

## Background

Patients with chronic obstructive pulmonary disease (COPD) are afflicted by a combination of emphysema in the peripheral lung and chronic obstruction in conducting airways, while asthma is predominantly acutely obstructive. According to the GOLD criteria, a post-bronchodilator FEV_1_/FVC-ratio below 0.7, which defines chronic airflow limitation (CAL), is required to make the diagnosis [1]. Additional factors need to be considered, but CAL is the only mandatory physiological requirement to establish a COPD diagnosis. While there is no similar requirement for asthma, an increase in FEV_1_ post-bronchodilation or significant variation in FEV_1_ over time are indicative of the disease [2]. Changes in FEV_1_, FVC, and their quotient are primarily indicators of obstruction, and can be caused by a number of factors, including inflammation, mucus hypersecretion, and hyperreactivity, which primarily affect conducting airways. In COPD, the lungs are also often affected by emphysema, with the severity varying greatly between patients.

Most conventional methods to measure lung function poorly reflect processes in the distal airspaces, as lung function measurements are typically most sensitive to the larger airways. To detect emphysema, imaging techniques and diffusing capacity of carbon monoxide (*D*_LCO_) are currently among the most reliable methods. However, *D*_LCO_ is not specific to emphysema, and imaging can be costly, time consuming, and could involve radiation exposure.

Our group has developed the Airspace Dimension Assessment (AiDA) technique, which is a novel method to measure the average radii of airspaces distal to the terminal bronchioles, including the alveoli [3–5]. This method utilizes the tendency of nanoparticles to deposit in the distal airspaces, the rate of which depends directly on the average distance between airspace walls. The airspace dimensions measured by AiDA have been shown to correlate strongly with the apparent diffusion coefficient obtained from diffusion-weighted MRI with hyperpolarized xenon gas [6], and to emphysema as assessed by computed tomography [7]. Previous work has shown that the lung properties measured with AiDA cannot be easily assessed with conventional pulmonary function tests [8].

While AiDA has been shown to accurately determine average distal airspace size, another parameter obtained from the AiDA analysis, the zero-second recovery (*R*_0_), is not as well understood. *R*_0_ is theorised to reflect the particle deposition that occurs during inhalation and exhalation and how the aerosol spreads throughout the lung [4]. Consequently, it has been assumed to contain information about the small conducting airways [4, 8]. Together, *r*_AiDA_ and *R*_0_ have the potential to indicate structural abnormalities in both the distal and the proximal lung. By investigating asthmatics, where we expect to find abnormalities in the conducting airways, and subjects with CAL, where we expect a heterogenous group with abnormalities in both the conducting and the respiratory regions, we can better evaluate the potential clinical use of AiDA and investigate whether it could be used to separate distal from proximal lung disease.

The objective of this study is to explore the potential of AiDA to rapidly determine the type and degree of obstruction in airway disease, and to separate it from emphysema. We investigate AiDA data from a subgroup of a population-based study of almost 700 people, whereof some have asthma or chronic airflow limitation, and evaluate whether patterns in *r*_AiDA_ and *R*_0_ could be used to separate the emphysematous component from the obstructive component in CAL as well as from asthma.

## Methods

### Study population

This work was performed as an add-on to the Swedish CArdioPulmonary bioImage Study (SCAPIS), which is a multi-centre population-based collaboration between six Swedish universities [9]. Men and women aged 50–64 years were enrolled from the Swedish population register. Enrolment was based on random selection, and no exclusion criteria were applied except for the inability to understand written and spoken Swedish for informed consent. Participants underwent conventional pulmonary function tests, computed tomography of the lungs, and answered a lifestyle and health questionnaire.

**Fig. 1:**
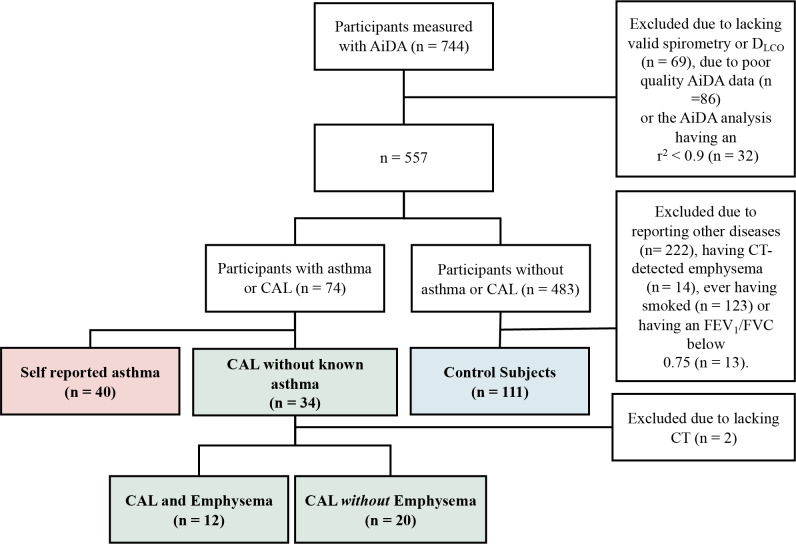
Flow chart of inclusion and exclusion. AiDA: Airspace Dimension Assessment; CAL: Chronic airflow limitation (a FEV_1_ / FVC ratio below 0.7)

Out of the 6251 participants enrolled in Malmö, 744 subjects were randomly selected to undergo AiDA examination [7, 8]. As is shown in Figs. 1 and 69 of these participants were excluded from the present study due to lacking valid diffusing capacity of carbon monoxide (D_LCO_) or spirometry data. Quality checks were established for AiDA to exclude measurements that were affected by technical issues such as low or unstable nanoparticle generation, as described in more detail by Petersson-Sjögren et al. [8]. Data from 86 subjects were excluded due to not passing these checks. Furthermore, a coefficient of determination (r^2^) below 0.9 for repeated AiDA samples for the same person also indicates a low-quality measurement and was reason for the exclusion of another 32 participants.

Out of 557 participants, 111 participants were selected as healthy controls. These were those who self-identified as healthy on the participant questionnaire, never smoked, had no detectible emphysema and a post-bronchodilation FEV_1_/FVC ratio of more than 0.75, as a ratio below this has been suggested to be indicative of asthma [10].

Participants who reported having asthma on the questionnaire were included in the asthma group (*n* = 40). Participants *without* self-reported asthma with an FEV_1_/FVC quotient below 0.7 post-bronchodilation were included in the chronic airflow limitation (CAL) group (*n* = 34). In the asthma group, 11 participants also met the requirements for CAL, and one was also diagnosed with COPD.

The participants meeting the CAL criteria were further subdivided into a group with emphysema (*n* = 12) as detected by computed tomography (CT), and one without (*n* = 20). Two subjects in the CAL group did not undergo a CT scan and were therefore not included in either subgroup.

### Lung function and imaging

All pulmonary function measurements were performed in accordance with the American Thoracic Society and European Respiratory Society standards [11, 12]. Spirometry (Jaeger MasterScreen PFT, CareFusion, Hoechberg, Germany) was performed 15 min after salbutamol-induced bronchodilation and FEV_1_ and FVC were obtained. Diffusing capacity was measured using a single-breath carbon monoxide diffusion test (Jaeger MasterScreen PFT) and the diffusing capacity of carbon monoxide (D_LCO_), the carbon monoxide transfer coefficient (K_CO_) and the alveolar volume (V_A_) were obtained. Spirometry and D_LCO_ were evaluated against the normal values in the SCAPIS cohort [13, 14]. Impulse oscillometry (IOS, Jaeger MasterScreen IOS, CareFusion, Hoechberg, Germany) was performed according to recommendations by Oostveen et al. [15], but with only a single measurement per subject. The resistance at 5 and 20 Hz (R5 and R20) were acquired, alongside the reactance at 5 Hz (X5) and the area of reactance (AX). Normal values for IOS were also calculated using reference equations acquired from the larger SCAPIS cohort (Table S1 in the supplementary material) [16].

All chest CTs in this study were performed using the same multidetector-row scanner (Siemens Somatom Definition Flash; Siemens Healthineers, Forchheim, Germany), with the methodology having been described in detail elsewhere [17, 18]. The imaging terminology was based on suggested terminology by the Fleischner Society [19]. Here, presence of emphysema was defined as CT findings of at least mild emphysema in any location by one of three experienced radiologists. In addition to radiologist-assessed emphysema, percentage of low attenuation volume (LAV%) and the 15th Percentile Density (PD15) were also acquired.

### Airspace dimension assessment (AiDA)

Distal airspaces are measured by analysis of inhaled and exhaled concentrations of 50 nm aerosol particles. The deposition fraction of this aerosol is calculated from inhaled and exhaled particle concentrations and relates directly to the size of the peripheral airspaces [4, 6]. The deposition fraction over several breath-holds is used to calculate an average airspace radius (*r*_AiDA_) and a zero-second recovery (*R*_0_).

The breathing manoeuvre during an AiDA measurement is similar to that used for single-breath D_LCO_, with the subject breathing into a mouthpiece while wearing a nose-clip. The subjects breathed particle-free air for approximately 30 s, after which air was exhaled to residual volume and then aerosol particles were inhaled to total lung capacity. Thereafter, the subjects held their breath for a few seconds and then exhaled. The exhaled aerosol concentration in exhaled air was measured after washout of dead space. The manoeuvre was repeated with breath-holds of 5, 7 and 10 s, with two successful replicates of each, for a total of 6 measurements.

**Fig. 2:**
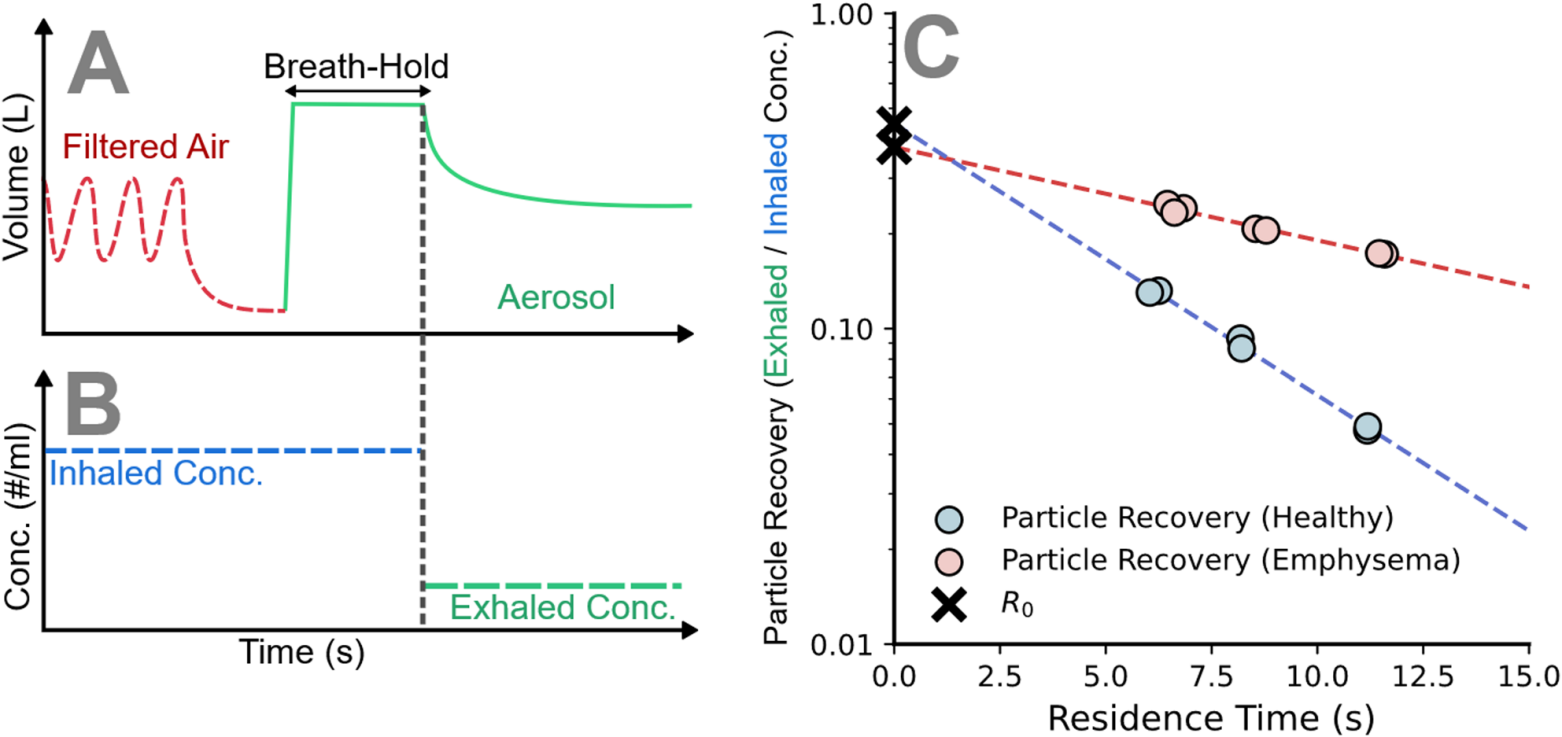
Principle behind the airspace dimension assessment, showing the measurement of respiratory flow (**A**), the concurrent measurement of aerosol concentration (**B**) and examples of the particle recovery measurements at different breath-hold times for a healthy subject and for one with emphysema (**C**). The half-life of the aerosol (t_½_) and the zero-second recovery (R_0_) are obtained from a regression of these points. In the emphysematous lung, the aerosol deposits at a slower rate than in the healthy lung (**C**)

The manner in which data is acquired by AiDA is shown in Fig. 2, and has been described in more detail elsewhere [3]. Briefly, 50 nm polystyrene latex spheres were aerosolized using an electrospray aerosol generator (Model 3480, TSI Inc., Shoreview, MN) and thereafter size selected using a differential mobility analyser (Model 3071, TSI GmbH, Aachen, Germany). Inhaled and exhaled nanoparticle number concentrations were determined by a condensation particle counter (Model A20, Airmodus Oy, Helsinki, Finland). The distal airspace radius, *r*_AiDA_, was calculated from the nanoparticle diffusion coefficient for 50 nm particles and the half-life (t_½_) of the inhaled particles, available from a least-squares regression of the particle recovery as a function of the nanoparticle residence-time in the lung, as seen in Fig. 2c. The *R*_0_ is the extrapolation of this regression to a theoretical residence-time of zero seconds.

### Statistical methods

The Shapiro-Wilk test revealed that *r*_AiDA_ was not normally distributed in any groups. Furthermore, sensitivity analysis of variable associations showed that the significance of parametric tests was driven by a few extreme values in lung function or AiDA values. Due to this, group comparisons and associations between variables were investigated by non-parametric tests. Differences between groups were evaluated using Kruskal-Wallis followed by pairwise Mann-Whitney comparisons. Associations between variables were evaluated using Spearman correlation. Calculations were performed using Python 3.10.7 (Python Software Foundation).

## Results

**Fig. 3:**
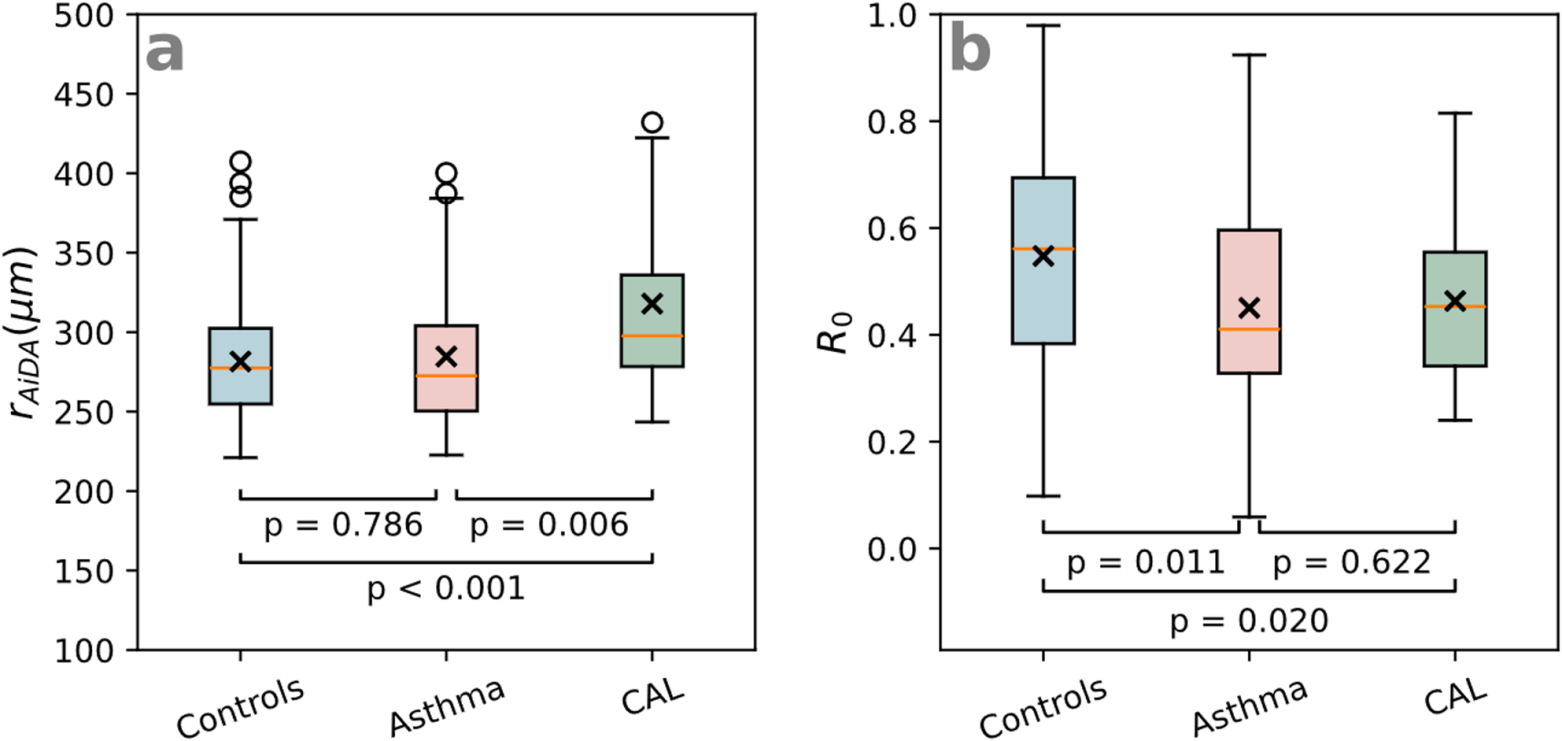
**a**, **b**: Boxplots of distal airspace radius (*r*_AiDA_, **a**) and zero-second recovery (*R*_0_, **b**) for the three groups. Lines represent the median, crosses the mean and circles mark statistical outliers. One outlier with 560 μm in *r*_AiDA_ is not visible in the CAL group in 3a. CAL: Chronic airflow limitation

Subject characteristics are shown in Table 1. As is shown in Table 2 and in Fig. 3, the median *r*_AiDA_ was approximately 13% larger in the group with CAL compared to that of the control group, while no significant change was seen in asthmatics. The median *R*_0_ was decreased by 20% in CAL and by 27% for asthmatics. All groups displayed closely comparable measurement quality, suggesting that there was no difference in adherence to the AiDA breathing manoeuvre or other systematic differences.

**Table 1:** Participant characteristics, morbidities and self-reported symptoms

	Controls (*n* = 111)	CAL (*n* = 34)	Asthma (*n* = 40)
Male / Female (*n*)	53 / 58	17 / 17	12 / 28
Age (years)	56 ± 4	58 ± 5	59 ± 4
Height (cm)	173 ± 10	172 ± 9	168 ± 8
Weight (kg)	80 ± 15	78 ± 14	77 ± 15
Pack-years	0	21 (0–36)	7 (0–14)
Emphysema (n)	0	12	7
Asthma (n)	0	0	40
COPD (n)	0	4	1
Cough (n)	11	12	15
Wheezing (n)	1	11	15
Dyspnoea on mild exertion (n)	8	10	15

Data are presented as number (n), means and standard deviations, or median and inter-quartile range

*CAL* Chronic Airflow Limitation, *COPD* Chronic Obstructive Pulmonary Disease

**Table 2:** Lung function, computed tomography, and AiDA data for the controls subjects and the groups with chronic airflow limitation (CAL) and asthma

	Controls (*n* = 111)	CAL (*n* = 34)	Asthma (*n* = 40)
FEV_1_ (% of predicted)	100.5 (91.5-107.1)	76.6 (63.8–87.6)***	87.5 (75.6–95.1)***††
FVC (% of predicted)	96.8 (90.5-103.6)	92.1 (79.0-104.9)	91.1 (83.6-100.2)*
FEV_1_/FVC	0.81 (0.79–0.84)	0.66 (0.63–0.69)***	0.76 (0.69–0.80)***†††
D_LCO_ (% of predicted)	96.0 (92.0-104.0)	84.7 (69.4–94.4)***	94.4 (84.9-101.1)†
K_CO_ (% of predicted)	103.2 (95.7-110.7)	84.4 (72.5–97.2)***	99.1 (91.3-107.7)††
V_A_ (% of predicted)	95.5 (89.9-101.2)	99.2 (89.8-103.7)	95.0 (86.7-105.1)
R5 (% of predicted)	105.8 (91.5-127.2)	122.5 (105.7-141.9)**	108.3 (92.3-139.8)
R20(% of predicted)	108.8 (94.5-131.1)	120.2 (107.0-142.1)*	111.9 (91.6–140.0)
X5 (% of predicted)	97.7 (80.8-113.3)	136.8 (98.1-160.8)***	126.9 (102.3-166.4)***
AX (% of predicted)	84.7 (59.0-120.0)	163.2 (83.9-249.1)***	149.7 (89.6-243.2)***
LAV (% volume)	4.6 (2.5–7.6)	7.5 (4.8–13.7)***	5.7 (3.5–10.0)
PD15 (HU)	-922 (-933–-909)	-934 (-948–-920)**	-927 (-939–-909)
*r*_AiDA_ (µm)	278 (255–302)	298 (278–336)***	273 (250–304)††
*R*_0_	0.56 (0.38–0.69)	0.45 (0.34–0.55)*	0.41 (0.33–0.60)*

Data are presented as medians and inter-quartile range

* *p *<0.05 compared to controls; ** *p *<0.01 compared to controls; *** *p* <0.001 compared to controls, † *p *<0.05 compared to CAL; †† *p* <0.01 compared to CAL; ††† *p* <0.001 compared to CAL. For R5, R20, and X5, two values are missing in the control group, one value is missing in the CAL group and one in the asthma group. For LAV% and PD15 one value is missing from the control group and two are missing from the CAL group

**Fig. 4:**
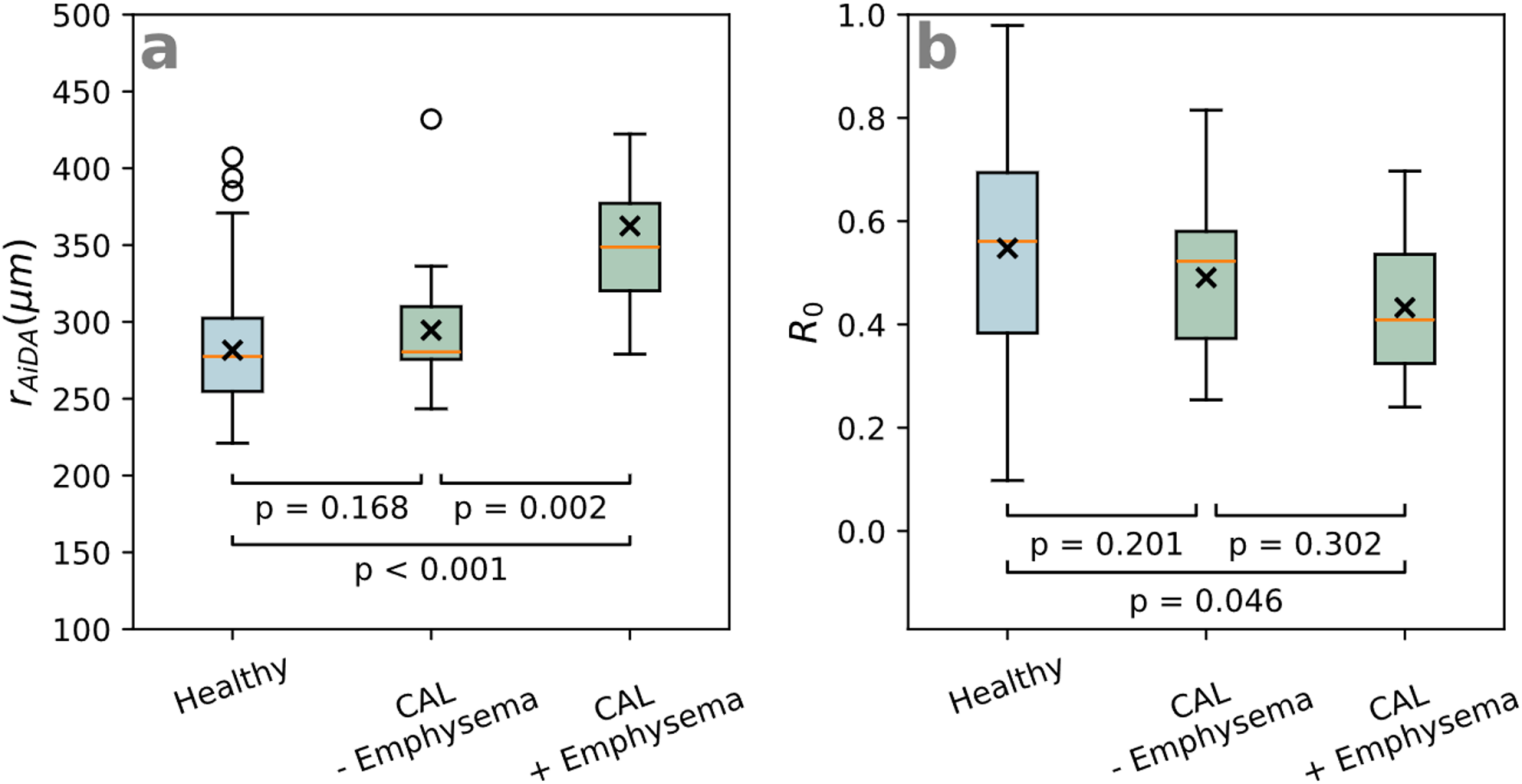
**a**, **b**: Boxplots of distal airspace radius (*r*_AiDA_, a) and zero-second recovery (*R*_0_, b) for the control group and the two CAL subgroups, with (n = 12) and without (n = 20) CT-detected emphysema. Lines represent the median, crosses the mean and circles mark statistical outliers. One outlier with 560 μm in *r*_AiDA_ is not visible in the CAL + emphysema group in 3a. CAL: Chronic airflow limitation

As seen in Fig. 4; Table 3, the 12 subjects with emphysema in the CAL group displayed further differentiation from the controls with a median *r*_AiDA_ 26% larger than the median control subject (*p* < 0.001). Conversely, the 20 subjects without CT-diagnosed emphysema displayed no significant increase in median *r*_AIDA_ when compared to control subjects (*p* = 0.168). The difference in *R*_0_ between control subjects and emphysematous CAL is marginally significant (*p* = 0.046), but no statistically different difference was found between the control subjects and non-emphysematous CAL. The difference in *r*_AIDA_ between CAL with emphysema and the asthmatic group was significant (*p* = 0.0004).

**Table 3:** Lung function and AiDA data of control subjects and the chronic airflow limitation (CAL) subgroups with and without CT-detected emphysema

	Controls (*n* = 111)	CAL + Emphysema (*n* = 12)	CAL - Emphysema (*n* = 20)
FEV_1_ (% of predicted)	100.5 (91.5-107.1)	67.2 (43.8–84.0)***	80.8 (73.1–88.5)***
FVC (% of predicted)	96.8 (90.5-103.6)	88.4 (73.0-105.0)	94.9 (87.0-105.0)
FEV_1_/FVC	0.81 (0.79–0.84)	0.61 (0.51–0.67)***	0.68 (0.65–0.69)***††
D_LCO_ (% of predicted)	96.0 (92.0-104.0)	68.2 (50.7–80.0)***	90.3 (81.8–97.9)*††
K_CO_ (% of predicted)	103.2 (95.7-110.7)	72.6 (60.0-81.6)***	89.9 (80.9-100.4)***††
V_A_ (% of predicted)	95.5 (89.9-101.2)	93.7 (87.0-103.8)	100.0 (96.4-104.4)*
R5 (% of predicted)	105.8 (91.5-127.2)	147.3 (120.6-171.9)***	111.4 (97.8-128.9)††
R20(% of predicted)	108.8 (94.5-131.1)	146.0 (110.3-151.1)**	116.0 (98.7-128.6)†
X5 (% of predicted)	97.7 (80.8-113.3)	153.0 (119.7-235.7)***	112.8 (84.7-144.9)†
AX (% of predicted)	84.7 (59.0-120.0)	245.4 (143.1-680.4)***	143.6 (64.5-211.8)†
LAV(% volume)	4.6 (2.5–7.6)	12.2 (5.0-15.7)**	7.0 (4.8–10.3)*
PD15 (HU)	-922 (-933–-909)	-944 (-952–-920)**	-929 (-941–-921)*
*r*_AiDA_ (µm)	277 (255–302)	349(320–377)***	281 (276–310)††
*R*_0_	0.56 (0.38–0.69)	0.41 (0.32–0.54)*	0.52 (0.37–0.58)

Data are presented as medians and inter-quartile range

* *p* <0.05 compared to controls; ** *P* <0.01 compared to controls; *** *p* <0.001 compared to controls, † p<0.05 compared to CAL with emphysema; †† p<0.01 compared to CAL with emphysema; ††† p<0.001 compared to CAL with emphysema. For R5, R20, and X5, two values are missing in the control group, one value is missing in the CAL group

The seven asthmatics *with* evidence of emphysema at CT had a median *r*_AiDA_ of 338 (328–373) µm and a median *R*_0_ of 0.33 (0.27–0.46); while the remaining 33 asthmatics had a median *r*_AiDA_ of 268 (249–280) µm and a median *R*_0_ of 0.42 (0.33–0.61). For the 5 people with diagnosed COPD, the median *r*_AiDA_ was 338 (335–418) µm and *R*_0_ was 0.50 (0.33–0.52). The 11 participants who reported asthma and met the criteria for CAL had a slightly increased *r*_AiDA_ of 292 (261–350) µm compared to controls, and decreased *R*_0_ of 0.35 (0.33–0.48). Removing the subjects with a COPD diagnosis from the CAL group lowered the median r_AiDA_ slightly, from 298 to 293 μm, but the result was still significantly different from controls (*p* = 0.009). Removing the subjects with COPD caused no change in the median *R*_0_ and only a minor change in significance level (*p* = 0.025). 

We found that self-reported symptoms were generally associated with an increased *r*_AiDA_ in CAL, but not in asthma. In the group with CAL, *r*_AiDA_ was significantly larger in participants reporting cough than in those not reporting cough (349 (311–403) vs. 282 (276–320) µm, *p* = 0.003). The trends were similar but not statistically significant for wheeze (336 (279–384) vs. 296 (278–330) µm, *p* = 0.33) and for dyspnoea during mild exertion (349 (317–375) vs. 293 (278–323) µm, *p* = 0.085). We saw no associations between symptoms and *R*_0_. 

In the CAL group (Table S3 in the supplementary material), there were significant correlations between *r*_AiDA_ and D_LCO_ (ρ=-0.46, *p* = 0.007) as well as K_CO_ (ρ=-0.60, *p* < 0.001). We also found a significant correlation between *R*_0_ and V_A_ (ρ = 0.37, *p* = 0.030). In the asthma group (Table S4 in the supplementary material), we saw a correlation between *r*_AiDA_ and K_CO_ (ρ=-0.53, *p* < 0.001) and V_A_ (ρ = 0.43, *p* = 0.006). We also saw correlations between *R*_0_ and FEV_1_ (ρ = 0.51, *p* < 0.001) and FVC (ρ = 0.50, *p* < 0.001).

## Discussion

This study demonstrates that Airspace Dimension Assessment (AiDA) detects increased distal airspace radii in chronic airflow limitation (CAL) and reduced zero-second recovery in both CAL and asthma. AiDA thus distinguishes emphysema and obstruction from normal lung function, with *r*_AiDA_ effectively identifying emphysematous individuals among those with chronic obstruction. The decrease in *R*_0_ in both asthma and CAL further supports its role as a marker of conducting airway dysfunction.

Separating asthma from chronic obstruction, and emphysema in particular, can be difficult. In this study, only D_LCO_ and AiDA showed a clear difference between the asthmatics and the CAL group. This is to be expected, as the breakdown of tissue, which leads to a decreased gas exchange rate, also results in an increased airspace size. However, D_LCO_ is an indirect measure of emphysema, and an isolated reduction in D_LCO_ can be indicative of vascular disease, interstitial lung disease, occupational exposure, or hypertension, among others [20, 21]. Meanwhile, *r*_AiDA_ should strictly be affected by the average airspace size and could therefore be more specific to emphysema than D_LCO_.

We show that, using AiDA, emphysematous individuals with CAL can be separated from those affected primarily by obstruction. This distinction is clinically relevant, as emphysema is associated with a less favourable prognosis than obstruction alone [22–24]. It is well known that emphysema may occur in smokers prior to airflow obstruction [25]. This condition is now recognised as pre-COPD [1]. Early recognition of emphysema may open for early intervention and improved prognosis. The AiDA technique can potentially be available even in out-patient clinics and may serve as a tool for early detection of emphysema. The method has several advantages. It is fast and easy for the patient to perform, with a breathing manoeuvre similar to measurement of D_LCO_. It provides a quantitative result that can be related to predicted values and it is conceptually easy to understand. In contrast to AiDA, D_LCO_ reflects not only alveolar size but also the diffusion across the alveolar-capillary barrier, vascular abnormalities and the availability of haemoglobin. Visual analysis of CT is observer dependent and quantitative analysis is affected by poor standardisation and reconstruction parameters. In this study, simple quantitative imaging measures such as LAV% and PD15 were able to separate healthy control subjects from subjects with CAL, but not from emphysema (as determined by a radiologist) from obstruction. Nor were these measures able to separate asthma from CAL. Somewhat surprisingly, *r*_AiDA_ seemed to reflect the radiologist’s assessment of emphysema to a higher degree than either LAV% or PD15.

We see a significant decrease in the FEV_1_/FVC quotient in both the asthma and the CAL groups, but only the CAL group displayed an increased *r*_AiDA_, which suggests that *r*_AiDA_ is not influenced by obstruction. Additionally, individuals with emphysema according to CT had a median *r*_AiDA_ of 349 μm, while subjects without emphysema were indistinguishable from controls. We can therefore say that obstruction does not influence *r*_AiDA_ to a significant degree. These trends are not seen for *R*_0_, where asthmatics as well as emphysematous and non-emphysematous CAL all display a decrease. Indeed, those with both conditions had among the lowest *R*_0_ values, with a median of 0.35. In contrast to *r*_AiDA_, this seems to indicate that *R*_0_ does reflect obstruction or alterations in the conducting airways.

We have previously seen that alpha-1 antitrypsin deficiency is linked to an increase in *r*_AiDA_, but not in *R*_0_, supporting the idea that these two parameters are independent of each other, and that *r*_AiDA_ reflects emphysema and *R*_0_ reflects abnormalities in the small conducting airways (manuscript in preparation). Similarly, in a previous study on preterm-born adolescents, *r*_AiDA_ was significantly larger than in control subjects, but no difference was observed in *R*_0_. This is thought to reflect a persistent underdevelopment of the alveoli in this group, while the conducting airways are unaffected [26].

The physiological reasons behind variations in *R*_0_ are not well understood, but since it represents the projected deposition in the airways at a residence time of zero seconds, it should reflect the particle deposition which occurs in the respiratory tract during convective transport of the inhaled aerosol. Intuitively, one might imagine that obstruction would cause more deposition due to the airways being narrower. However, this is not the case for smooth-walled tubes, as the increased velocity of the air offsets the shorter distance that the particle must travel to deposit. Hence, deposition probability is independent of tube diameter. Instead, we suggest that it might be an effect of increased airway surface-roughness and local turbulence. It has been shown theoretically that particle deposition by diffusion is increased in proportion to the degree of “unevenness” of the airways [27]. A rougher structure might be the result of airway thickening, hyperplasia, mucus hypersecretion, or a combination of larger surface area and bronchoconstriction.

Consistent with our findings, Wang et al. showed, using hyperpolarized Helium-3 diffusion-weighted MRI, that the apparent diffusion coefficient was more than doubled in COPD patients compared to controls, while in asthmatics the increase was smaller [28]. In that study, the increase in apparent diffusion coefficient in asthmatics was attributed to air-trapping, which is not expected to have as great an effect on *r*_AiDA_ (although it might have an effect on *R*_0_), as we expect nanoparticles to mainly enter ventilated regions of the lung. As with *r*_AiDA_, the increase in apparent diffusion coefficient for COPD patients is attributed to emphysema. Emphysema has also been associated with increased airspaces when measured using *aerosol-derived airway morphometry* [29]. Aerosol-derived airway morphometry, like AiDA, utilizes the properties of aerosol to determine size of the distal airspaces, but necessitates a more challenging inhalation manoeuvre and requires prior knowledge of total lung capacity [4].

All lung function measurements, including AiDA, were performed after bronchodilation, and it is possible that the results would have been further differentiated from control subjects if the measurements had been done without bronchodilation, particularly for asthmatics. Despite the large size of the cohort, the number of subjects with CAL or asthma were limited. Larger sample sizes would have increased the statistical robustness of our findings. In addition, while the CAL group is unambiguous, the asthma group relies on self-reporting, which makes it less reliable. We did not apply corrections for multiple comparisons. However, given the limited number of groups, the consistency of our findings with previous studies, and concerns about potential overcorrection, we consider this approach to be justified. While the AiDA technique is no more challenging for the patient than other lung function tests, the current method of generating the nano-aerosol requires training of the operator. However, recent developments will likely allow for improved usability in the future [30].

This is the first study which shows that AiDA can be used to separate emphysematous from primarily obstructive lung disease. We also see a similar decrease in zero-second recovery in subjects with chronic airflow limitation and asthma, which further strengthens the idea that this value reflects conducting airway dysfunction, and consequently, that the airspace radius measured by AiDA is not affected by obstruction. With further development, it is possible that AiDA could be used as a method to separate emphysema and obstruction and consequently aid in phenotyping COPD and asthma.

## Supplementary Information

Supplementary Material 1.

## Data Availability

Because of the sensitive nature of the personal data and study materials, they cannot be made freely available. However, by contacting the study organization (www.scapis.org ; Email: scapis@scapis.org), procedures for sharing data, analytic methods, and study materials for reproducing the results or replicating the procedure can be arranged following Swedish legislation.

## Abbreviations

COPD: Chronic obstructive pulmonary disease
CAL: Chronic airflow limitation
AiDA: Airspace Dimension Assessment
GINA: Global Initiative for Asthma
GOLD: Global Initiative for Chronic Obstructive Pulmonary Disease
SCAPIS: the Swedish CArdioPulmonary bioImage Study
CT: Computed tomography
r_AiDA_: Distal airspace radius
R_0_: Zero-second recovery
FEV_1_: Forced Expiratory Volume in one second
FVC: Forced Vital Capacity
D_LCO_: Diffusing capacity of the lungs for carbon monoxide
K_CO_: Carbon monoxide transfer coefficient
V_A_: Alveolar volume
IOS: Impulse oscillometry
R5: Resistance at 5 Hz
R20: Resistance at 20 Hz
X5: Reactance at 5 Hz
AX: Area of reactance
LAV%: Low attenuation volume, as percent of lung volume
PD15: 15th Percentile Density
HU: Houndsfield units
