# Supplementary material for: Aerosol deposition in the lung as a biomarker in asthma and chronic obstruction
Source: Respir Res. 2026 May 11;27:224. doi: 10.1186/s12931-026-03706-2 (PMC13248413; doi:10.1186/s12931-026-03706-2)
Supplement: Supplementary file 1 — Supplementary Material 1. [file 12931_2026_3706_MOESM1_ESM.docx]

**Supplementary Material**

**Table S1**: Medians (50^th^ percentiles) for R5, R20, X5, AX, F_res_. A: age; H: height in cm; W: weight in kg.

| **Variable** | **Reference equation** |
| --- | --- |
| R5 Male (kPa s L^-1^) | 0.9650 – 0.0006*A – 0.0048*H + 0.0022*W |
| R5 Female (kPa s L^-1^) | 0.9458 – 0.0004*A – 0.0045*H + 0.0020*W |
| R20 Male (kPa s L^-1^) | 0.7111 – 0.0007*A – 0.0030*H + 0.0011*W |
| R20 Female (kPa s L^-1^) | 0.6467 – 0.0007*A – 0.0023*H + 0.0008*W |
| X5 Male (kPa s L^-1^) | -0.2836 – 0.0000*A + 0.0014*H – 0.0004*W |
| X5 Female (kPa s L^-1^) | -0.4124 – 0.0001*A + 0.0023*H – 0.0008*W |
| AX Male (kPa L^-1^) | 0.8988 + 0.0001*A – 0.0054*H + 0.0022*W |
| AX Female (kPa L^-1^) | 1.6015 + 0.0004*A – 0.0104*H + 0.0046*W |
| F_res_ Male (Hz) | 27.6349 + 0.0100*A – 0.1393*H + 0.0692*W |
| F_res_ Female (Hz) | 32.0529 + 0.0070*A – 0.1674*H + 0.0798*W |

**Table S2:**  Spearman’s correlation coefficient (ρ) and the associated p-value between lung function and AiDA variables (*r*_AiDA_ and *R*_0_) in the group with chronic airflow limitation (CAL).

| Variable | *r*_AiDA_ |  | *R*_0_ |  |
| --- | --- | --- | --- | --- |
|  | **ρ** | **p-value** | **ρ** | **p-value** |
| FEV_1_ (% of predicted) | -0.012 | 0.946 | 0.108 | 0.542 |
| FVC (% of predicted) | 0.102 | 0.565 | 0.172 | 0.331 |
| FEV_1_ /FVC | -0.299 | 0.086 | -0.026 | 0.882 |
| D_LCO_ (% of predicted) | -0.456 | 0.007 | -0.073 | 0.680 |
| K_CO_ (% of predicted) | -0.605 | <0.001 | -0.237 | 0.178 |
| V_A_ (% of predicted) | 0.124 | 0.486 | 0.373 | 0.030 |
| R5 (% of predicted) | 0.192 | 0.286 | -0.154 | 0.391 |
| R20 (% of predicted) | 0.225 | 0.208 | -0.176 | 0.327 |
| X5 (% of predicted) | -0.031 | 0.864 | -0.167 | 0.353 |
| AX (% of predicted) | -0.067 | 0.713 | -0.142 | 0.429 |
| LAV% | 0.279 | 0.122 | 0.077 | 0.673 |
| PD15 | -0.244 | 0.178 | -0.055 | 0.764 |
| *r*_AiDA_ |  |  | 0.330 | 0.057 |
| *R*_0_ | 0.330 | 0.057 |  |  |

**Table S3:**  Spearman’s correlation coefficient (ρ) and the associated p-value between lung function and AiDA variables (*r*_AiDA_ and *R*_0_) in the group with asthma.

| Variable | *r*_AiDA_ |  | *R*_0_ |  |
| --- | --- | --- | --- | --- |
|  | **ρ** | **p-value** | **ρ** | **p-value** |
| FEV_1_ (% of predicted) | 0.087 | 0.592 | 0.505 | <0.001 |
| FVC (% of predicted) | 0.390 | 0.013 | 0.503 | <0.001 |
| FEV_1_ / FVC | -0.200 | 0.216 | 0.029 | 0.859 |
| D_LCO_ (% of predicted) | -0.249 | 0.121 | 0.102 | 0.532 |
| K_CO_ (% of predicted) | -0.527 | <0.001 | -0.179 | 0.270 |
| V_A_ (% of predicted) | 0.427 | 0.006 | 0.328 | 0.039 |
| R5 (% of predicted) | 0.132 | 0.415 | -0.120 | 0.461 |
| R20 (% of predicted) | 0.122 | 0.454 | -0.098 | 0.548 |
| X5 (% of predicted) | -0.005 | 0.978 | -0.167 | 0.304 |
| AX (% of predicted) | -0.006 | 0.971 | -0.130 | 0.424 |
| LAV% | 0.458 | 0.003 | 0.255 | 0.112 |
| PD15 | -0.422 | 0.007 | -0.277 | 0.083 |
| *r*_AiDA_ |  |  | -0.028 | 0.865 |
| *R*_0_ | -0.028 | 0.865 |  |  |

**Table S4:**  Spearman’s correlation coefficient (ρ) and the associated p-value between lung function and AiDA variables (*r*_AiDA_ and *R*_0_) in the group of healthy control subjects.

| Variable | *r*_AiDA_ |  | *R*_0_ |  |
| --- | --- | --- | --- | --- |
|  | **ρ** | **p-value** | **ρ** | **p-value** |
| FEV_1_ (% of predicted) | 0.243 | 0.010 | 0.159 | 0.095 |
| FVC (% of predicted) | 0.224 | 0.018 | 0.101 | 0.292 |
| FEV_1_ / FVC | -0.086 | 0.370 | 0.029 | 0.764 |
| D_LCO_ (% of predicted) | -0.140 | 0.143 | -0.234 | 0.013 |
| K_CO_ (% of predicted) | -0.419 | <0.001 | -0.387 | <0.001 |
| V_A_ (% of predicted) | 0.278 | 0.003 | 0.203 | 0.032 |
| R5 (% of predicted) | 0.106 | 0.271 | -0.037 | 0.702 |
| R20 (% of predicted) | 0.102 | 0.290 | -0.041 | 0.673 |
| LAV% | 0.295 | 0.002 | 0.222 | 0.020 |
| PD15 | -0.309 | 0.001 | -0.210 | 0.028 |
| *r*_AiDA_ |  |  | 0.146 | 0.126 |
| *R*_0_ | 0.146 | 0.126 |  |  |
